# Competency-Based Training and Assessment of Listening Skills: A Waitlist-Controlled Study in European Telephone Emergency Services

**DOI:** 10.32872/cpe.7933

**Published:** 2022-12-22

**Authors:** Simone Jennissen, Stefan Schumacher, Diana Rucli, Melinda Hal, András Székely, Derek de Beurs, Ulrike Dinger

**Affiliations:** 1 Department of General Internal Medicine and Psychosomatics, University Hospital Heidelberg, Heidelberg, Germany; 2 Department of Psychosomatic Medicine and Psychotherapy, Medical Faculty, Heinrich Heine University Düsseldorf, Düsseldorf, Germany; 3 TelefonSeelsorge Hagen-Mark, Hagen, Germany; 4 Studio Rucli Formazione e Consulenza organizzativa, Udine, Italy; 5 Department of Applied Psychology, Faculty of Health Sciences, Semmelweis University, Budapest, Hungary; 6 Szent Rókus Hospital, Psychiatry, Baja, Hungary; 7 Végeken Egészséglélektani Alapítvány, Budapest, Hungary; 8 Trimbos-instituut, Utrecht, The Netherlands; Philipps-University of Marburg, Marburg, Germany

**Keywords:** listening skills, training, telephone emergency services, helpline, paraprofessional counselors

## Abstract

**Background:**

Telephone emergency services (TES) provide an essential part of suicide prevention and emotional support services across different health care settings. TES are usually provided by paraprofessional counselors, who need specific training in listening skills to meet the demands of callers.

**Method:**

This project developed a competency-based training for listening skills which was then evaluated in a randomized controlled waitlist study across four EU countries (Germany, Hungary, Italy, and the Netherlands). Each country provided one training group and one waitlist group. Across countries, a total of 71 (trained: n = 36, waiting: n = 35) counselor trainees were assessed in a standardized, simulated emergency call with an actor client either before or after training participation. Calls were audiotaped and competencies in listening skills were evaluated by external raters using a standardized rating form.

**Results:**

Trained counselors showed significantly better listening skills than participants from the waitlist condition.

**Conclusion:**

Results provide support for the efficacy of a competency-based training for listening skills in the field of TES across Europe. Furthermore, results demonstrated that a standardized competency-based assessment with an actor client is suitable to assess listening skills.

## Highlights

- A competency-based training can improve paraprofessionals’ listening skills in a relatively short training time.
- Listening skills can be assessed in a simulation with an actor client.
- The use of competency-based training and assessment methods could be expanded to the field of paraprofessional counseling.

Telephone Emergency Services (TES) form an important part of psychosocial health care, emotional support services, and suicide prevention (Dinger et al., 2019). TES are usually free of charge, available at all times, and do not require help-seeking individuals to disclose their identity. Thus, there is a small barrier for those in need to reach out to TES. This is also represented in the number of calls TES receive. In 2019, the German TES *TelefonSeelsorge* responded to 1.2 million calls (Telefonseelsorge, 2019). Similarly, the Australian *Lifeline* reports over one million calls yearly (Lifeline, 2020), the United Kingdom’s *Samaritans* reported over 3.6 million calls in 2018 (Samaritans, 2019), and the United States’ *National Suicide Prevention Lifeline* reported more than 22 million calls in 2018 (The National Suicide Prevention Lifeline, 2019), which underlines the widespread acceptance and need for TES. During the COVID-19 pandemic, TES have gained even more importance since there were both needs for social distancing as well as increased mental health burdens. TES responds well to both needs as a low-threshold mental health service that can be accessed even by high risk patients during times of rigorous infection control measures (Arenliu et al., 2020; Humer et al., 2021; Kavoor et al., 2020).

As opposed to psychotherapists, psychiatrists, and social workers who participate in year-long professional training curricula before providing mental health services, TES counselors are paraprofessionals with limited and regionally different training. A study conducted on the German *TelefonSeelsorge* showed that TES counselors receive training over the course of seven to 24 months (*M* = 13.3 months; Dinger & Rek, 2017). The *Samaritans*’ conduct their training in five to ten sessions over the course of a few months (Samaritans, 2020). Despite having no formal medical or psychological education, TES counselors frequently deal with highly stressed callers. In 2019, 43.7% of callers in Germany presented suicidal thoughts, 6.6% stated an intent to commit suicide, and 7.1% had formerly attempted suicide (Telefonseelsorge, 2019). Most callers repeatedly contacted TES for emotional support, which could be an indicator of high mental strain. Frequently discussed topics included experiencing depression or anxiety, interpersonal difficulties, or physical health issues (Telefonseelsorge, 2019). Studies from the United Kingdom (Coveney et al., 2012), the United States (Ingram et al., 2008; Mishara et al., 2007), and Australia (Burgess et al., 2008) report similar contents. As such, the topics discussed in TES calls are comparable with the contents of psychotherapy sessions, despite TES counselors receiving far less formal training for handling difficult clients.

Training is important not only to provide adequate service to callers, but also for the well-being of TES counselors themselves. In a meta-analytic review, Hattie et al. (1984) showed that the amount of training that paraprofessionals received was associated with their effectiveness as counselors on a variety of outcome measures such as clients’ self-reported change, clinical ratings by independent raters, information provided by significant others, work performance, or therapist improvement ratings. Paraprofessionals with “some experience” (e.g. hospital workers, medical students, or speech pathologists) were more effective than inexperienced paraprofessionals (e.g. college students, volunteer adults). A more recent review on the effectiveness of professional and paraprofessional counselors to deliver cognitive-behavioral treatment for depression and anxiety also concluded that training is important for paraprofessional counselors to deliver effective service (Montgomery et al., 2010). Furthermore, a qualitative survey suggests that paraprofessional counselors wish for more training in order to feel confident in dealing with difficult clients (Skoglund, 2006). Studies on psychotherapists show that training increases therapists’ self-efficacy (Hess et al., 2006; Pascual-Leone & Andreescu, 2013). Note that while skills are defined as the ability to carry out an activity and competencies additionally include the knowledge of when and how to apply one’s skills, self-efficacy encompasses one’s *confidence* in one’s own capabilities, but not actual skills or competencies (Bandura, 1977; Butler, 1978; Le Deist & Winterton, 2005). However, evidence from a systematic review suggests that counselor self-efficacy is related to counselor performance as assessed by trained raters and supervisors (Larson & Daniels, 1998). Thus, training is necessary to both directly increase counselors’ efficacy as well as to boost their confidence in their own capabilities. Within TES, as there are large numbers of callers and limited resources, paraprofessionals’ training is distinctively shorter than professionals’ training.

Since TES are local organizations without uniform training standards, there is a need for more research on time-efficient, focused training opportunities that equip volunteer counselors with the key competencies they require. Listening skills form an integral part of many counselor trainings and are the core of TES trainings (Hill, 2009; Ivey et al., 1987). They comprise a variety of techniques such as active listening, showing empathy, supporting clients’ self-efficacy, establishing rapport with the client, and exploring feelings of the client (Hill, 2009; Rogers & Farson, 1957). Listening skills may rather be categorized as competencies, since they also include the knowledge about when and how to apply a specific skill and refer to the broader concept of being able to listen to, soothe, and help another person (Butler, 1978; Le Deist & Winterton, 2005). However, since listening *skills* is an established term, this term will be used throughout the paper.

This study aimed to develop and evaluate a competency-based training for listening skills. To account for the heterogeneity of TES and extend the generalizability of our results, the study was conducted as an international multisite project in Germany, Italy, Hungary, and the Netherlands. Furthermore, while research in psychotherapy and counseling mostly relies on self-report measures, these are likely biased due to limited introspectiveness of respondents. Counselors, for instance, might over- or underestimate their skills depending on their level of self-criticism (Anderson et al., 2016). In psychotherapy research, recent studies have therefore employed competency-based assessments of therapist skills, such as the Facilitative Interpersonal Skills (FIS) performance test (Anderson et al., 2009). The FIS is used to assess therapists’ interpersonal behavior in a standardized test situation. Therapists are asked to respond to challenging therapy situations that are presented to them either as video clips or with actor clients. Therapists’ responses are filmed and later evaluated by trained judges according to a rating manual (Munder et al., 2019). In this study we intended to employ a competency test methodology similarly to the FIS. Specifically, we aimed to assess listening skills in a simulated TES call with an actor representing a typical TES client. As in the FIS, trained judges evaluate participants’ listening skills based on recordings of the simulated calls using a standardized rating sheet. This allows a more objective assessment of paraprofessional counselors’ listening skills in an ecologically valid setting, while also directly assessing the competencies needed in a TES call. We hypothesized that trained participants would demonstrate better listening skills in the standardized simulated emergency call than participants who had not received the listening skills training.

## Method

The Ethics Committee (Institutional Review Board) of the department of psychology at Heidelberg University approved the study procedures (reference number: AZ Jenn 2020 1/1). Participants were informed about all study procedures by the local member of the research team and provided informed consent prior to participation.

### Participants and Procedure

The study was designed as a randomized-controlled waitlist trial. Participants were recruited at local TES posts in Germany, Hungary, Italy, and the Netherlands via participating institutions in the Erasmus+ funded network EmPoWEring (Educational Path for Emotional Well-Being). As a widely known organization, TES posts are regularly contacted by individuals who are interested in becoming a volunteer counselor for TES. During our study period from November 2016 to April 2017, those who contacted TES about becoming a volunteer counselor were informed about the study and the opportunity to participate in the listening skills training. Those consenting to the study procedures were then cluster-randomized within site to start training either immediately (training group) or delayed (waitlist group). Within each country, the research team randomized each individual to either an immediate training group or a waitlist group. Participants in the training group immediately started the listening skills training. After the training groups had completed their training, listening skills of participants in both training and waitlist groups were assessed in a standardized, simulated emergency call with an actor client. After the assessment, the waitlist group received their listening skills training. Due to the naturalistic recruitment, there is no information available on the number of individuals who decided against participating in our study. There were no dropouts after enrollment.

Participants had to be 18 years or older to be eligible. A total of *N* = 71 volunteer counselors (*n* = 12 from Germany, *n* = 20 from Hungary, *n* = 20 from Italy, and *n* = 19 from the Netherlands) participated in our study. Each country provided on training group and one waitlist group. Across countries, a total of *n* = 36 participants were randomized to the training group and *n* = 35 were randomized to the waitlist group. The majority of participants (82%) were female. Participants’ mean age was 38.51 years (*SD* = 15.86). About half of the sample (48%) reported a school diploma and 52% a university degree as their highest level of education. Participants were asked whether they had prior work experience as a “listener”, either volunteering for a counseling or emergency service or as a professional therapist or counselor before participating in this study. About half (45%) of participants reported prior professional or voluntary work experience as a listener for a mean duration of 6.96 years (*SD* = 8.76). Descriptive characteristics by group (training vs. waitlist) are presented in Table 1. There were no significant differences between study groups regarding descriptive characteristics.

**Table 1:** Descriptive Characteristics for the Training and Waitlist Group

Characteristic	Training group *n* = 36		Waitlist group *n* = 35		Difference test	
	*M*	*SD*	*M*	*SD*	*t*	*p*
**Age**	40.1	15.7	36.9	16.1	-0.848	.400
**Former experience in listening (years)**	2.4	6.0	3.8	7.5	0.832	.408
	***N***	**%**	***N***	**%**	**χ^2^**	***p***
Gender					2.53	.112
Male	4	11.1	9	25.7		
Female	32	88.9	26	74.3		
Highest educational level					1.283	.733
Basic secondary school	5	13.9	7	20.0		
High school	12	33.3	10	28.6		
Bachelor’s degree	10	27.8	12	34.3		
Master’s degree	9	25.0	6	17.1		
Former experience in listening					0.137	.712
Yes	17	47.2	15	42.9		
No	18	52.8	20	57.1		

*Note*. Former experience in listening refers to prior work experience as a “listener”, either volunteering for a counseling or emergency service or as a professional therapist or counselor before participating in this study.

### Listening Skills Training

A focus group of professionals in TES counseling and pastoral care developed a manual for the listening skills training. The 120 hr training is split into three parts: a 30 hr self-study online module to convey the theoretical basis of listening, a 40 hr practical group training in listening which is provided in 10 structured sessions, and a 50 hr module for in-depth practice and supervised training calls. Table 2 provides a more detailed overview of the training modules. Participants’ attendance was monitored for all in-class events and there were no missed sessions. Attendance of the self-study online module was not assessed by the research team.

**Table 2:** Description of Contents of the Listening Skills Training Modules

Module	Content
1. Self-study (30 hrs)	Using an e-learning tool, participants are provided with 100 multiple choice questions regarding the theoretical basis of listening. After each question, participants receive feedback on their selected answer(s) and are presented with a brief theoretical explanation. Topics include cognitive-behavioral, psychodynamic, systemic, and humanistic/client-centered theories.
2. Practical group training (40 hrs)	This part of the training is performed on site in groups of maximum 15 participants.
Session 1: Introduction	focuses on a personal introduction of group members, self-reflection of training goals and motivations, and the assessment of existing knowledge and views on listening
Session 2: Active Listening	teaches the principles of active listening (how to ask for thoughts/feelings/behaviors, give the other person space, and paraphrase meaningful contents)
Session 3: Emotional stability	teaches ways to regulate one’s own and the other person’s feelings
Session 4: Respect and boundaries	fosters acceptance of differences between people teaches ways to set boundaries in the listening process
Session 5: Empathy	fosters perspective taking and empathic responses to another person’s story
Session 6: Mirroring	teaches ways to reflect the other person’s feelings or statements
Session 7: Self-reflection	encourages reflection on own feelings, motivations, and resources
Session 8: Structuring conversations	teaches the five-phase model of the listening process (welcome, exploration, goal setting, elaboration, conclusion)
Session 9: Strengths and resources	teaches how to ask for resources and foster strengths of the other person
Session 10: Feedback and conclusions	summarizes acquired listening skills and encourages reflection on personal progress
3. In-depth practice (50 hrs)	Having acquired the theoretical knowledge as well as practical experience in role plays and group exercises, the final part of the listening skills training is focused on supervised training cases. This module should be adapted to suit the needs of listeners in their specific work environment.

### Assessment

Listening skills were assessed in a standardized, simulated emergency call with a trained actor client. The actor role represented a typical TES caller. Actors received a standardized role script with a detailed description of their role as well as instructions for a 15-minute TES call. There was one native speaking actor in each country. Before the assessment, actors prepared their role and practiced the simulated call with paraprofessional counselors of different experience levels. This ensured that actors were trained to respond realistically to a variety of possible interventions by participants. Furthermore, these practice calls were recorded and used as training material for the observer ratings of listening skills. During the assessment period, a local member of the research team listened to recordings of the standardized, simulated emergency call and gave feedback regarding role adherence to the trained actor client on a weekly basis.

Assessments were conducted by telephone to mimic a naturalistic TES setting. Calls were recorded for assessment purposes. Participants were called by blinded research assistants and instructed to be a good listener for an actor client for about 15 minutes. After assuring that the instructions were clear, the actor then took over the phone and presented herself as “Laura”, a 27-year-old office clerk, who was struggling in her relationship and also stressed out by her current job workload. “Laura” was calling TES when she was home alone in the evening and overwhelmed by her feelings. She was severely distressed, but not in an acute suicidal crisis. “Laura” was struggling to identify her own emotions, but she was willing to respond to the paraprofessional counselor’s questions and able to benefit from the listening process.

Listening skills were assessed using an observer rating measure. The Listening Skills Scale (LSS) was developed by members of the research team (SJ, UD) based on several validated psychotherapy process scales, i.e. the Multitheoretical List of Therapeutic Interventions (MULTI; McCarthy & Barber, 2009), the Active Empathetic Listening Scale (AEL; Drollinger et al., 2006), the Working Alliance Inventory (WAI-SR; Hatcher & Gillaspy, 2006), and the Therapist Empathy Scale (TES; Decker et al., 2014) and adopted the methodology of the FIS performance test (Anderson et al., 2009). Items were modified to suit the TES environment (i.e. “client” instead of “patient”; “listener” instead of “therapist”) and to reflect an observer perspective. The scale consisted of 33 items representing listening skills such as perspective taking, respect, active listening, resource activation, and structuring the conversation. Higher values represent better listening skills. Items include “The listener sometimes finds it difficult to see things from the other person’s point of view *(inversed)*” or “The listener appreciates their client as a person”. Items are evaluated on a 5-point Likert scale (1 – *totally disagree*; 5 – *totally agree*) with one additional *N/A* category in case an item cannot be assessed from the information in the audio recording of the standardized simulated emergency call. Two items are reverse coded. Higher values represent better listening skills. Internal consistency of the scale was excellent in the present study (Cronbach’s α = .94). The full scale is available in the online supplement.

Ratings were provided by at least on trained research assistant in each country. Recordings of practice calls from the actor training were used to train raters in the application of the LSS. During the assessment period, at least once per week the local member of the research team listened to recordings of the standardized, simulated emergency calls, gave feedback to the actor (see above), and supervised the local research assistant in ratings on the LSS. In the German subsample, all LSS ratings were performed by two independent observers. Interrater reliability of these two raters was excellent, ICC(3,1) = .86.

### Data Analytic Strategy

As a first step, we explored missing data and investigated the factor structure of the listening skills scale as a basis for further analyses. We performed a principal component analysis (PCA) using the Scree criterion for factor retention to determine whether calculating a mean score for listening skills was appropriate. Next, we assessed whether our data was normally distributed. Since each of the four countries provided one training group and one waitlist group, groups were nested within country. We therefore assessed whether this introduced dependency in our data by calculating the intraclass correlation (ICC) within countries in a multilevel intercept only model. We intended to employ a multilevel model to assess group differences if there were an ICC ≥ .05. An ICC < .05 would indicate that country does not affect outcome and therefore single level multiple regression models would be appropriate (Tabachnick & Fidell, 2014). We employed a stepwise modeling procedure. The first model tested for group differences in listening skills without covariates. To assess the robustness of results, the second model introduced age and gender as common covariates and the third model adjusted for years of previous experience as a listener outside of the TES environment. Effect sizes were calculated as standardized regression coefficients. A standardized regression coefficient of *b* = .10 is considered small, *b* = .30 is considered moderate, and *b* = .50 is considered large (Cohen, 1988).

## Results

### Preliminary Analyses

Missing data analysis demonstrated more than 5% missing values in six items of the LSS. We therefore excluded these items from the following analysis.

Next, we conducted a principal component analysis (PCA) to explore the factor structure of the LSS. The Kaiser-Meyer-Olkin score of KMO = .86 and the significant Bartlett’s test of sphericity, χ^2^(351) = 1562.15, *p* < .001, demonstrated the adequacy of the data for PCA. The Scree plot was slightly ambiguous and showed inflexions that would justify both retaining one or two components. Inspections of the factor loadings indicated a higher-order general factor of “listening skills” which explained 48.58% of variance. We therefore decided to retain one component and calculate a mean value for listening skills as a basis for further analyses. Factor loadings are available in the online supplement. Based on a visual inspection of the histogram, negligible skew (-0.18) and kurtosis (-0.54), as well as a nonsignificant Kolmogorov-Smirnov test (*p* = .20), listening skills were normally distributed across participants.

### Effect of the Listening Skills Training

**Figure 1:**
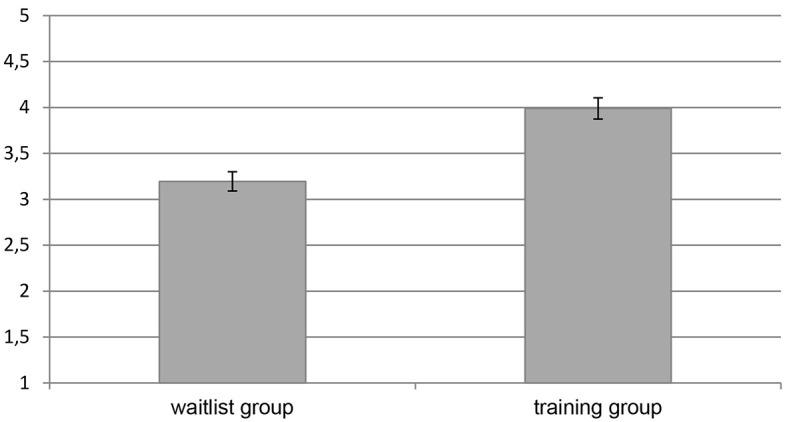
Mean Listening Skills of Participants in the Training Group and the Waitlist Group *Note. N* = 71 (*n* = 36 participants were randomized to the training group and *n* = 35 were randomized to the waitlist group). Error bars represent the standard error of the mean. Listening skills were assessed in a standardized, simulated emergency call using the observer-rated Listening Skills Scale (LSS). Scale values range from 1-5, where higher values indicate better listening skills. The difference between the groups is significant (*p* < .05), see result of the linear regression model in Table 3.

Since groups were nested within countries, we first assessed the dependency in our data by calculating the ICC within countries in a multilevel intercept only model. With an estimated ICC of .01, the model suggested negligible dependency in the data. Hence, multiple regression was deemed an appropriate method to test for group differences. The first model predicted listening skills as measured by the LSS from group (waitlist group vs. training group). Group was a significant predictor of listening skills with a large standardized regression coefficient of b* = .52 (see Table 3). Participants in the training group (*M* = 3.99, *SD* = 0.69) demonstrated significantly better listening skills than participants in the waitlist group (*M* = 3.20, *SD* = 0.62, see Figure 1). To assess the robustness of this effect, we next employed a hierarchical model introducing age and gender as covariates in the first step and group in the second step. While there was no significant effect of age or gender, group remained as a predictor of listening skills with a large standardized regression coefficient of b* = .54 (see Table 3). Lastly, we assessed whether previous experiences in listening affected the observed listening skills. The final hierarchical model introduced years of previous experiences in listening outside of TES in the first step and group in the second step. Age and gender as nonsignificant predictors were dropped from this model. There was no significant effect of previous experience, while group continued to significantly affect listening skills with a large standardized regression coefficient of b* = .52 (see Table 3).

**Table 3:** Linear Regression Models Predicting Listening Skills

Parameter	Model 1		Model 2		Model 3	
	Coefficient (*SE*)	95% CI	Coefficient (*SE*)	95% CI	Coefficient (*SE*)	95% CI
Intercept	3.20 (0.11)*	[2.97, 3.42]	3.46 (0.27)*	[2.93, 4.00]	3.20 (0.12)*	[2.96, 3.45]
Age			-0.01 (0.01)	[-0.02, 0.00]		
Gender			-0.06 (0.21)	[-0.48, 0.35]		
Experience					-0.00 (0.01)	[-0.03, 0.02]
Group	0.79 (0.16)*	[0.48, 1.11]	0.82 (0.16)*	[0.50, 1.14]	0.79 (0.16)*	[0.47, 1.11]
Model Fit						
*R* ^2^		0.27		0.28	0.27	
Adjusted *R*^2^		0.26		0.26	0.25	

*Note. N* = 71; Gender was dummy coded (0 – male, 1 – female). Experience = years of previous experience in listening outside of telephone emergency services. Group was dummy coded (0 – waitlist group, 1 – training group). Listening skills were assessed in a standardized, simulated emergency call using the observer-rated Listening Skills Scale (LSS).

*p < .05.

## Discussion

This study aimed to develop and evaluate a competency-based training for listening skills in an international multisite project across Europe. Results provide support for the efficacy of the 120 hr training. Trained individuals demonstrated significantly better listening skills than their untrained counterparts. The effect size for this group difference was large, which implies that this relatively short training makes a meaningful difference in paraprofessional counselors’ abilities to adequately respond to TES calls. Furthermore, the effect of the training was independent from participants’ age, gender, and previous experience as a listener in other contexts. Although approximately half of the participants reported previous experiences in the field of “listening”, e.g. in their profession as social workers, nurses, or pastoral care workers, or as a volunteer for other services, these experienced participants benefitted as much from the training as inexperienced participants. This implies that the training is suitable for groups with different levels of expertise and equips paraprofessional counselors with specific competencies needed within TES. Listening on the telephone may require a different set of skills than listening in a face-to-face setting, such as the ability to fully rely on verbal expressions in understanding the client, without the option to consider nonverbal cues (Sötemann, 2019). The counselors themselves also have to convey their interest in the client, their caring and respectful attitude, and the comfort they provide solely through speech and voice modulation. Silence, which could serve a holding function in a face-to-face setting, might feel uncomfortable or even threatening to a client on the phone who has no means to determine whether the counselor is still with them. Lastly, the anonymity of TES could be unfamiliar to those who have never worked in listening of the phone and make it difficult to build a relationship at the beginning (Sötemann, 2019). These differences between face-to-face and telephone settings might explain while experiences in listening outside of the TES environment were not an advantage in our study and experienced participants also needed the training to acquire the specific competences needed to adequately respond to a TES call.

In this study, the assessment of listening skills was realized with an actor patient in a simulated emergency call. This method was chosen not only for a more objective assessment, independent of participants’ ability to accurately report on their own listening skills, but also to tap into the exact competencies needed for the later task as a paraprofessional counselor in TES. Competency-based assessment methods have gained increased popularity in medical education and psychotherapy over the last decades (Anderson et al., 2016; Dannefer & Henson, 2007; Lurie, 2012). They are based on the insight that neither factual knowledge, nor self-evaluation are sufficient to guarantee the mastery of a practical task (Miller, 1990). To assure that trainees can perform their tasks competently, assessments should be performed in the context of the actual workplace or in a realistic simulation (Holmboe et al., 2010; Issenberg et al., 2005). Thereby, the assessment can include context factors from the real life setting and confirm that trainees are prepared for authentic encounters. The employed assessment method of a standardized, simulated emergency call with an actor client fulfilled these requirements. Participants were presented with a typical TES caller and could therefore demonstrate their competency as a paraprofessional counselor in TES. The assessment showed that the training sufficiently teaches listening skills as they a required in everyday practice at TES.

### Limitations

This study is limited in generalizability by the recruited sample. Although we performed the study as a multisite project across four different European countries, TES operate internationally, and future studies will determine whether the listening skills training is effective in other than the investigated countries. However, investigating the training across four countries with very different local structures (Germany, Italy, Hungary, and the Netherlands) is a major strength of this study and the focus on European countries seems sensible since a large number of TES sites operate in Europe (IFOTES, 2020). Another limitation of this study is the small sample size within each country. Although the achieved power to detect the overall group difference was ≈ 1 (Faul et al., 2007), drawing statistical inferences at the country level would have proven difficult. However, by calculating the ICC we assured that outcomes did not differ depending on the country in which participants were assessed.

Next, although actors received a detailed role script, prepared their role thoroughly, and were trained and supervised frequently, the actors had to react flexibly to participants’ interventions and therefore the assessment was not completely standardized. Future studies could investigate whether presenting pre-recorded audio sequences is a viable alternative, although this comes at the cost of a less ecologically valid assessment situation.

Furthermore, although participants received a standardized training of 120 hrs in total, their attendance in the 30 hr online module was not monitored by the research team and thus may have varied. Further evaluations of the training should assess attendance in all modules and control for missed classes in statistical analyses.

Next, though reliability measures within this study demonstrated excellent interrater agreement and internal consistency of the LSS, further validation of the scale, preferably with listening skills measures from different perspectives, would be useful.

Lastly, due to limited resources we designed the study as a randomized controlled waitlist trial with a single assessment in each group. Assuming randomization was successful, this procedure should result in correct effect size estimates for the training. However, a baseline assessment in the training group could have been used to examine the successfulness of randomization and could also have served as a more direct measure of existing knowledge than asking for previous experiences in listening. Furthermore, future evaluations of the listening skills training may want to include a follow-up assessment to examine long-term effects of the training.

### Implications and Conclusion

Our findings have several implications. First and foremost, demonstrating the efficacy of the training in participants from several European countries suggests that the listening skills training can be used to train paraprofessional counselors at TES from different countries. The modular structure allows for flexibility while also providing an evaluated and effective basis. International TES sites may use the listening skills training as a basic curriculum and adapt it to their regionally different needs. To monitor their trainees’ development of competencies, they could also make use of the assessment method with the standardized acting role. Although role-plays are typically part of the TES group training, introducing a standardized assessment could help trainers and trainees identify their specific needs while also providing a consistent background against which paraprofessional counselors’ listening skills can be evaluated.

Furthermore, the increased demand for mental health services during the COVID-19 pandemic together with the necessity to reduce in-person contact between individuals has highlighted two core competencies of TES: they are widespread available and offer emotional support in a socially distant manner (Humer et al., 2021; Kavoor et al., 2020). Although trainings such as the helping skills training or postgraduate training programs for psychotherapists, psychiatrists, and social workers are well-established (Hill, 2009; Hill & Lent, 2006), the current rapid increase in demand for mental health services underlines the usefulness of short, effective trainings for listening skills.

Lastly, this study aimed to evaluate the use of competency-based training and assessment methods in the field of paraprofessional counseling. Although commonly accepted as beneficial in medical education (Lane et al., 2001; Scalese et al., 2008), competency-based methods are still rare in the field of psychotherapy and counseling. Similarly to simulation patients in medical education, this study introduced an assessment with a standardized actor client to a paraprofessional counseling environment. Future studies should investigate the use of an actor client to assess counseling competencies in the field of professional counseling and psychotherapy.

To conclude, this international multisite study demonstrated the efficacy of a competency-based training for listening skills across Europe. Trainees successfully acquired listening skills in the 120 hr course, as demonstrated in a standardized simulated emergency call with an actor representing a typical TES caller. Findings encourage the application of the training in TES to prepare volunteers for their tasks as paraprofessional counselors. Furthermore, results suggest that competency-based assessment in a simulated TES call is a suitable method to measure listening skills.

## Supplementary Materials

Provides an observer-rating measure of listening skills (Listening Skills Scale). The Listening Skills Scale (LSS) was used by independent observers to rate listening skills of participants in simulated emergency calls (for access see Index of Supplementary Materials below).



JennissenS.
SchumacherS.
RucliD.
HalM.
SzékelyA.
de BeursD.
DingerU.
 (2022). Supplementary materials to "Competency-based training and assessment of listening skills: A waitlist-controlled study in European telephone emergency services"
[Measurement instrument]. PsychOpen. 10.23668/psycharchives.8308
PMC988112536762348
